# Xanthoceras Sorbifolium Bunge Oil: Extraction Methods, Purification of Functional Components, Health Benefits, and Applications in Production and Daily Life

**DOI:** 10.3390/foods14061004

**Published:** 2025-03-16

**Authors:** Can Cui, Yongrou Fang, Yujie Mu, Lian Yang, Longhao Zeng, Huihui Li, Huanjiang Wang, Lingyun Zhou, Chunyan Li, Yadian Xie

**Affiliations:** 1 School of Chemical Engineering, Guizhou Minzu University, Guiyang 550025, China; m17785023430@163.com (Y.F.); m15628003749@163.com (Y.M.); y15086290181@163.com (L.Y.); gzmzwhj@163.com (H.W.); zhoulingyun201508@163.com (L.Z.); cyli2023@gzmu.edu.cn (C.L.); 2 Monitoring Center of Ecological and Environmental of Guizhou Province, Guiyang 550081, China; zeng_lh@hotmail.com (L.Z.); gzslhh915@163.com (H.L.)

**Keywords:** Xanthoceras sorbifolium Bunge oil, extraction methods, bioactive functions, applications

## Abstract

Xanthoceras sorbifolium Bunge is an oil-bearing shrub native to China, whose seeds are rich in oil and can be used for extracting edible oil. The primary extraction methods for Xanthoceras sorbifolium Bunge oil (XSBO) include pressing, solvent extraction (SE), ultrasound-assisted extraction (UAE), aqueous enzymatic extraction (AEE), micro-wave-assisted extraction (MAE), and supercritical carbon dioxide extraction (SFE-CO_2_). This review not only compares the advantages and disadvantages of these oil extraction techniques regarding extraction principles, oil yield efficiency, and cost-effectiveness but also reviews the existing purification processes for the active components in oil. XSBO exhibits various health benefits, including antibacterial, antioxidant, anti-inflammatory, and antitumor properties. In particular, it contains a special component called nervonic acid, which rarely exists in other plant oils, and has garnered significant attention for its potential in alleviating the impact of neurological diseases. XSBO has been widely applied in food, pharmaceuticals, and health supplements. However, the underlying mechanisms of its bioactive functions have not been fully elucidated, and there is limited research on encapsulation techniques, which restricts its application in food and pharmaceutical health products. Further studies in this domain can focus on purification processes, identifying the precise mechanism of action, to achieve efficient development and utilization of XSBO.

## 1. Introduction

**Figure 1:**
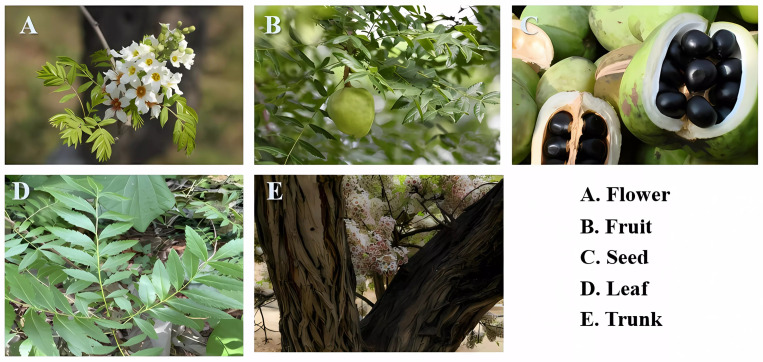
The morphological characteristics of Xanthoceras sorbifolium Bunge.

Xanthoceras sorbifolium Bunge (XSB), commonly referred to as Wen-guan-guo or Wen-deng-guo (Figure 1), is an endemic oil-bearing shrub belonging to the Sapindaceae family in China, often recognized as the “northern oil-tea” [1]. XSB exhibits a well-developed root system and demonstrates low environmental requirements, thriving in nutrient-poor soils. Particularly in arid and semi-arid regions, it effectively mitigates soil erosion and serves as a keystone species for desertification control and ecological rehabilitation. The plant significantly contributes to ecosystem stabilization through these attributes. Its prolonged flowering period (approximately 20 days) provides sustained nectar resources for pollinator communities, including Apis species, thereby contributing to the maintenance of ecosystem balance [2]. Additionally, the cultivation of XSB drives the development of related industries, including seedling cultivation, processing, and sales, creating employment opportunities. Moreover, due to its strong adaptability, it can be grown on marginal lands such as barren mountains and sandy areas, enhancing the economic productivity of such land.

The entire XSB is considered a valuable resource. In traditional Chinese medicine, in addition to its culinary uses [2], XSB is used to treat various conditions, including rheumatism and enuresis in children [3,4]. With the advancement of technology, many researchers have conducted chemical composition and functional studies on its leaves, husks, flowers, pericarps, and other parts [5,6]. For instance, the saponins extracted from the leaves of XSB demonstrate a significant inhibitory effect on Escherichia coli and Streptococcus, and thus can be utilized as natural antibacterial agents for food [7]. The triterpenoid saponin XS-8 contained in the fruit shells of XSB has anti-Alzheimer’s disease effects and can serve as a raw material for clinical drugs [8]. Additionally, the XSB flowers’ extract has shown inhibitory effects on cells of benign prostatic hyperplasia and possesses potential anti-cancer properties [9]. Furthermore, the coumarin-type component, odorin B, isolated from the seed coat of XSB, demonstrates strong anti-HIV-1 activity and has low toxicity to human T-lymphocytes [10].

Xanthoceras sorbifolium Bunge oil (XSBO) is primarily extracted from the seeds and kernels of XSB. The oil content in the seeds of XSB is approximately 35%, while that in the kernels is over 60%. XSBO predominantly comprises eight different fatty acids. Among them, the types and amounts of saturated fatty acids are relatively low, mainly including palmitic acid (C16:0) and stearic acid (C18:0). The content of unsaturated fatty acids ranges from 92.06% to 94.20%. In descending order of content, they are linoleic acid, oleic acid, erucic acid, arachidonic acid, palmitic acid, nervonic acid, stearic acid, and linolenic acid. Linoleic acid and oleic acid, which have the highest contents, account for 61.57–68.39% of the total amount of these eight main fatty acids and are the principal components of XSBO. In addition, the protein content ranges from 20.27% to 37.88% [11,12]. XSBO is characterized by its high content of unsaturated fatty acids, rich and high-quality proteins, and abundant active ingredients. It exhibits various beneficial functions, including lowering blood lipids, preventing cardiovascular diseases, exhibiting anti-inflammatory and antioxidant properties, providing neuroprotective effects, improving intestinal function, and promoting digestion and excretion [13,14]. From the perspective of edible applications, its oil is rich in unsaturated fatty acids, making it a high-quality edible oil that can meet the demands of a healthy diet, thereby demonstrating significant potential in the edible oil market. In the field of medical and health products, XSBO can be used to develop a variety of products, creating considerable economic value. Examples include “Linoleic Acid Dropping Pills”, “Yishouning”, and “Linoleic Acid Microcapsules” [15,16]. However, research on its applications in the cosmetics industry has not yet been reported. In the energy sector, it can serve as a raw material for biodiesel production, contributing to clean energy supply and alleviating energy pressure. Industrially, it can be used to manufacture products such as lubricants and paints.

Currently, research on XSBO primarily encompasses the fields of medicine, food, and industry (e.g., bioenergy, chemical industry, and so on). This article aims to provide a comprehensive review of the extraction methods, purification techniques, functional activities, and applications of XSBO as an industrial ingredient (e.g., edible oil, medical health products, biodiesel, lubricating oil, and so on), aiming to offer insights for more effective development and high-value utilization of this oil.

## 2. Extraction of XSBO and Purification of Its Functional Components

### 2.1. Extraction Methods

#### 2.1.1. Pressing

The pressing method refers to a method of squeezing oil out of oil-bearing materials with the help of mechanical external force. The pressing method can be divided into the cold pressing and hot pressing method, and the main difference between the two lies in whether the oil-bearing materials undergo a high-temperature treatment process before pressing. Deng et al. took the seeds of XSB as raw materials and obtained the cold-pressed oil of XSB by means of the mechanical pressing method. The results demonstrated that the optimal technological conditions for cold-pressed XSBO were as follows: the pressure was 55 MPa ± 2, the ratio of kernel to shell was 9:1 (g/g), the pressing time was 8 h, and the yield rate of XSBO reached 40.44% [17]. Furthermore, Li et al. optimized the extraction of XSBO by employing the microwave pretreatment pressing method. The results demonstrated that the optimal process conditions were as follows: the moisture content of the raw materials was 8.3%, the ratio of kernel to shell was 9:1, the microwave treatment time was 5.4 min, and the microwave power was 960 W. Under these process conditions, the oil yield of XSB was 56.79%. Compared with the oil yield (46.37%) of direct cold-pressed XSB, it increased by 10% [18]. Excessive pressing temperatures can lead to oil oxidation, affecting the nutrient content in XSBO, while temperatures that are too low may reduce the oil yield. Insufficient pressure results in incomplete oil extraction, whereas excessive pressure may compromise oil quality. If the pressing duration is too short, the oil yield will be low, but if it is too long, it may increase energy consumption and the risk of oil oxidation. The oil yield of the pressing method is influenced by multiple factors, including the raw material quality, pressing conditions, equipment type, and process optimization. By selecting high-quality raw materials, controlling the pressing temperature and duration, optimizing the equipment parameters, and incorporating auxiliary technologies, the oil yield of XSBO can be significantly improved while ensuring oil quality. Although the pressing method features a simple process and can preserve the nutrients and flavor of XSBO, the physical extrusion approach may also lead to a relatively low oil yield of XSB.

#### 2.1.2. Solvent Extraction (SE)

The principle of SE is solid–liquid extraction; the oil is transferred from the raw material to the solvent through diffusion, then the solvent is heated to evaporate. Finally, the crude oil undergoes refining to remove impurities, contaminants, and solvent residues. When selecting an extraction solvent, several factors should be considered: solvent cost and properties, environmental safety, and human toxicity. Among these, the most critical factor influencing oil extraction efficiency is solvent polarity. SE requires the use of large amounts of organic solvents, such as ethanol, acetone, pentane, hexane, chloroform, and ethyl acetate [19]. Lu et al. used cyclohexane as the extracting agent to investigate the impacts of the liquid-to-material ratio, extraction time, and extraction temperature on the extraction rate of XSBO. The results indicated that when the liquid-to-material ratio was 4:1, the extraction time was 3.0 h, and the extraction temperature was 70 °C, the extraction rate reached its maximum value of 30.02% [20]. Wang et al. used petroleum ether as the extracting agent. Under the conditions of an extraction temperature of 90 °C, a liquid-to-material ratio of 5:1, and an extraction time of 10 h, the extraction rate of XSBO was 62.49% [21]. The use of different extracting agents can result in significant variations in the oil extraction rate from XSB. To enhance the oil extraction rate, in addition to selecting an appropriate extracting agent, multiple extraction and reflux processes can also be employed. The extraction temperature significantly affects the oil yield. As the temperature rises, the extraction rate initially increases but then declines. Higher temperatures enhance molecular thermal motion, promoting interaction between the solvent and oil molecules, which facilitates the transfer of oil into the solvent and increases the extraction rate. But excessively high temperatures cause solvent evaporation and potential degradation of oil components, reducing the extraction efficiency. When the solvent-to-material ratio decreases (i.e., the amount of solvent increases), the oil yield significantly improves but stabilizes after reaching a certain point. Existing studies have not thoroughly investigated the impact of these factors on the oil composition [19,20,21]. In future research, on the one hand, efforts should be focused on analyzing the impact of the solvent extraction process on the components of XSBO. On the other hand, it is also necessary to consider the introduction of new alternative extraction agents. Future research should focus on optimizing the extraction process and analyzing its effects on oil components to enhance the industrial application potential of XSBO. SE is widely used due to its advantages such as short extraction time, high extraction efficiency, simple process, and low cost. However, this method requires the consumption of large amounts of organic solvents, and the resulting oil may contain residues of harmful reagents to the human body. Furthermore, prolonged extraction may degrade some lipophilic bioactive compounds or unsaturated fatty acids in the oil, leading to significant loss of the oil’s inherent aroma and flavor.

#### 2.1.3. Aqueous Enzymatic Extraction (AEE)

AEE destroys the oilseed through enzymatic hydrolysis and separates the oil and hydrophilic components with water, to achieve the simultaneous separation of oil, protein, etc. The main steps of AEE include the grinding and crushing of oily raw materials, enzymatic hydrolysis, centrifugation, and demulsification to recover oil. Due to the multi-chamber and multi-component structure of oilseed cells, it is recommended to use a combination of enzymes with different activities to disrupt oilseed cells. Selecting the appropriate enzyme or enzyme combination is a crucial step. The enzymes used in AEE mainly include cellulase, protease, phospholipase, and pectinase. Furthermore, factors such as the amount of enzyme and water, temperature, pH value, and processing time also have significant impacts on the extraction efficiency [22,23]. Wang et al. utilized the kernels of XSB as raw materials and applied AEE to extract XSBO. They examined the effects of enzyme types, enzyme dosages, solid–liquid ratios, enzymatic hydrolysis temperatures, and hydrolysis durations on the extraction rate of XSBO. The results demonstrated that the optimal process conditions were as follows: the solid–liquid ratio was 1:5, the enzymatic hydrolysis time was 5 h, the hydrolysis temperature was 50 °C, and the enzyme dosage was 1.5%. Under these optimal conditions, the average extraction rate of XSBO was approximately 65.10% [24]. Kang et al. carried out an in-depth exploration of the AEE of XSBO. Through comparative experiments, they determined that the extraction efficiency of neutral protease was the highest. The results from single-factor experiments and orthogonal experiments indicated that the optimal conditions for extracting XSBO using neutral protease were as follows: a temperature of 50 °C, an enzyme dosage of 3%, a moisture content of the material embryo of 12%, and a hydrolysis duration of 4 h. Under these specific conditions, the extraction rate of XSBO achieved 74.82% [25]. Liu et al. utilized three enzymes (Celluclast 1.5 L complex cellulase, Viscozyme L pentosan complex enzyme, and Alcalase 2.4 L hydrolytic protease) to perform oil extraction from the kernels of XSB. The results indicated that Alcalase 2.4 L protease exhibited superior performance among the three. Through a series of systematic experiments and optimizations, the optimal process conditions were established as follows: enzyme concentration, 2% (*v*/*m*); temperature, 55 °C; pH, 9; material-to-liquid ratio, 1:6 (*m*/*v*); and enzymatic hydrolysis time, 2 h. Under these precisely controlled conditions, the oil extraction yield reached 78.67%. Furthermore, AEE can be combined with other extraction methods [26]. For instance, Gu et al. took the kernels of XSB as raw materials and adopted the ultrasonic-assisted enzymatic method to extract the oil from XSB kernels. They investigated the impacts of the ultrasonic temperature, ultrasonic time, ultrasonic power, and enzyme dosage on the extraction rate of XSBO. The optimum conditions, with an actual yield of 71.5%, were as follows: 50 °C ultrasonic temperature, 20 min ultrasound time, 150 W ultrasound power, and 1000 U/g enzyme dosage [27]. Qiu et al. utilized the powder of XSB kernels as raw materials, pretreated the samples with the assistance of ultrasonic waves, and then adopted AEE to extract XSBO. They explored the influences of factors such as the enzyme types, enzymatic hydrolysis pH values, solid–liquid ratios, temperatures, and times on the extraction rate of XSBO. The results demonstrated that the optimal parameters were as follows: the solid–liquid ratio was 1:6 (*m*/*v*), the temperature was 45 °C, the dosage of alkaline protease (at a pH value of 7.0) was 3.0%, the dosage of cellulase (at a pH value of 4.5) was 1.0%, and the reaction time was 8 h (two enzymes were subjected to enzymatic hydrolysis for 4 h, respectively); under these conditions, the total extraction rate of free oil could reach 81.2% [28].

The influence of different enzymes on the oil extraction rate varies significantly. Enzymes such as cellulase, compound enzymes (cellulase and pectinase in a specific ratio), neutral protease, and Alcalase 2.4 L protease can all increase the oil extraction rate to varying extents. Among them, the compound enzyme and cellulase exhibit similar effects and are superior to pectinase, while neutral protease achieves a relatively high extraction rate [26]. Alcalase 2.4 L protease demonstrates remarkable efficacy and achieves optimal oil extraction performance at a specific dosage. As the enzyme dosage increases, the oil extraction rate initially rises and then stabilizes. Beyond a certain dosage, the oil extraction rate no longer improves significantly due to limitations in the enzyme’s degradation capacity and the content of the substrate. Within a certain range, increasing the liquid-to-solid ratio enhances the oil extraction rate, but exceeding the optimal ratio leads to a decline. This is because a low liquid-to-solid ratio results in insufficient contact between the substrate and enzyme, while a high ratio reduces the concentrations of both the substrate and enzyme, both of which impede oil extraction. As the enzymatic hydrolysis temperature rises, the oil extraction rate initially increases and then decreases. At low temperatures, enzyme activity is weak, resulting in a low oil extraction rate; as the temperature increases, enzyme activity strengthens, improving the oil extraction rate. However, excessively high temperatures cause enzyme denaturation, leading to a reduction in the oil extraction rate. In most studies, enzymatic hydrolysis temperatures within the range of 45–60 °C yield higher oil extraction rates [26,27]. Prolonging the enzymatic hydrolysis time initially increases the oil extraction rate. However, the rate then stabilizes or even declines. In the early stages, extended time allows more thorough interaction between the substrate and enzyme, increasing the oil extraction rate. However, excessive duration may lead to oil oxidation or equilibrium in the enzymatic reaction, preventing further increases or even reducing the oil extraction rate. Existing studies have not explored the effects of different enzymes under varying parameters on the composition of XSBO [25]. Future research directions should focus on optimizing the process and analyzing its impact on the components of the oil, so as to enhance the industrial application potential of XSBO. The AEE method is considered to be an extraction method based on the concepts of “green chemistry” and “green engineering” because of its no-solvent residue, environmental friendliness, and high oil yield. Furthermore, the extracted oil obtained by AEE has superior physicochemical parameters and nutrients compared to pressing and SE. However, due to the high equipment requirements, expensive enzymes, and complex process, the cost of AEE is relatively high, which may affect its economic benefits.

#### 2.1.4. Ultrasonic-Assisted Extraction (UAE)

UAE leverages the mechanical and cavitation effects within an ultrasonic system to disrupt the cell wall structure and reduce the particle size, thereby accelerating solvent penetration into oilseed cells and enhancing the oil extraction rate within a short period of time. The primary processes of UAE include solvent diffusion within the cell wall and the extraction of intracellular oil. The water content of the sample, the degree of grinding, and the type of solvent are significant factors influencing the extraction rate. Additionally, the temperature, pressure, ultrasonic frequency, and duration are crucial factors that affect the effectiveness of ultrasound. High temperatures aid in disrupting the interaction between the solvent and the matrix, increasing the solvent diffusion rate, while low temperatures are beneficial for enhancing cavitation erosion. Generally, the extraction rate increases as the time increases, but excessively long extraction durations may lead to undesirable changes in the system [29,30]. Chen et al. used petroleum ether as the extraction solvent and employed UAE to extract seed oil from XSB; the optimal conditions, with a single-pass oil yield of 58.95%, were as follows: an extraction temperature of 70 °C, the ultrasonic power was 150 W, the extraction time was 34 min, and the liquid-to-solid ratio was 7:1 (*v*/*m*) [31]. Fan et al. compared the extraction rates of oil from XSB seeds using the pressing, SE, and UAE methods. The results revealed that the oil yields for the pressing, SE, and UAE methods were 56.7%, 62.3%, and 68.5%, respectively. UAE produced an oil yield that was 11.8% and 6.2% higher than that of the pressing and SE methods, respectively. Additionally, UAE can be combined with other methods for extracting oil from XSB [32]. Liang et al. used XSB seeds as raw materials and employed an ultrasonic and NaCl demulsification-assisted enzymatic aqueous method to extract XSBO. The results showed that under the optimal conditions (150 W ultrasonic power, 120 min extraction time, 0.05% cellulase addition, 0.10% NaCl addition, 1:3.5 liquid-to-solid ratio, and 30 °C enzymatic hydrolysis temperature), the extraction rate of XSBO could reach 89.85% [33]. According to relevant studies, within the ultrasonic power range of 0–150 W, the oil yield of XSB increases with higher power, but decreases beyond 150 W [31]. Additionally, within a certain time range (which varies depending on specific conditions), the oil yield increases with prolonged ultrasonic time. This is because, in the initial stage, the oil content in the XSB kernel powder is high, and the significant concentration gradient between the solid and liquid phases provides a strong driving force for diffusion. As a result, a large number of oil molecules diffuse into the solvent, leading to a continuous increase in oil yield. However, beyond a certain time, as the oil content in the liquid phase increases and approaches saturation, the osmotic pressure of the system stabilizes, and the amount of oil continuing to dissolve becomes minimal. At this point, further prolonging the ultrasonic time has little to no effect on the oil yield and may even cause a decline. Currently, there are no reported studies on the effects of ultrasonic power on the composition of XSBO. However, theoretically, excessively long ultrasonic durations may induce oxidation or decomposition reactions in some components of the oil, thereby altering its composition. This is because ultrasound generates heat and mechanical effects during the process, and prolonged exposure may disrupt the molecular structure of the oil, potentially reducing the content of unsaturated fatty acids or causing other changes. Nevertheless, these hypotheses require further experimental validation. UAE has high efficiency, requires a short extraction time, and produces few extraction impurities that are easy to separate. But the main drawbacks of this method are as follows: the uneven distribution of ultrasonic energy, which leads to a decrease in extraction efficiency over time, and the inability to replace the solvent during the extraction process. Although UAE can slow down the oxidation rate under certain conditions, high-power ultrasonic treatment (>20 kHz) can have adverse effects on the quality of the oil (peroxide value, free acidity, total polar compounds, and fatty acid composition) [33].

#### 2.1.5. Microwave-Assisted Extraction (MAE)

Microwave radiation possesses unique penetration characteristics and can penetrate deeply into cells for heating. After the substances within the cells absorb microwave energy, they rapidly heat up and expand, exerting significant pressure on the cell walls. When this pressure exceeds the maximum threshold that the cell walls can endure, the walls rupture, causing the internal substances of the cells to leak out and come into full contact with the solvent. This enhances mass transfer efficiency and, consequently, effectively boosts the extraction rate of oil. Microwave technology is commonly used as a pretreatment to lyse cells, achieving the purpose of improving the extraction efficiency. The extraction efficiency of MAE is influenced by various factors, such as the microwave duration, power, temperature, frequency, and wavelength [34,35]. Zhang et al. employed the Negative Pressure Cavitation–Microwave Assisted Extraction (NMAE) technique to extract the seed oil of XSB. Under optimal conditions, the extraction yield of XSBO could reach 52 ± 1%. Additionally, they also investigated the extraction kinetics of both the NMAE and SE methods. The results indicated that the NMAE method was more efficient than the SE method [36]. Chen et al. used XSB seeds as raw material and employed MAE extraction to obtain XSBO. The optimal conditions were as follows: the extraction temperature was 75 °C, the extraction time was 17 min, the liquid-to-solid ratio was 20:1, and the microwave power (P) was 400 W. Under these process conditions, the extraction yield of XSBO was 58.85% [37]. Within a certain range, the oil yield of XSB gradually increases with the rise in the extraction temperature in MAE. This is because higher temperatures enhance the fluidity of organic solvents and internal oils in the plant, reducing viscosity and facilitating the extraction of oils by the solvent. As the microwave extraction time is extended, the contact time between the solvent and raw materials increases, leading to a higher oil yield. Similarly, within a certain range, increasing the liquid-to-solid ratio improves the oil yield, but beyond a certain point, the yield decreases. This is because insufficient solvent volume cannot effectively extract the target product, while excessive solvent increases microwave energy consumption and reduces the heating and extraction efficiency. The order of influence of the extraction parameters on the oil yield of XSB is as follows: extraction temperature > liquid-to-solid ratio > extraction time > microwave power. Different parameter conditions do not significantly affect the types of fatty acids, but the liquid-to-solid ratio has a significant impact on the content of unsaturated fatty acids. The higher the liquid-to-solid ratio, the greater the content of unsaturated fatty acids. Currently, there are few and insufficiently in-depth studies on the effects of microwave-assisted extraction on the composition of XSBO. Future research should focus on further optimizing the process and analyzing its impact on oil components. MAE boasts advantages such as low energy consumption, short extraction time, and reduced use of organic solvents. Microwaves rapidly increase the system temperature in a short period, lowering the viscosity of oils and fats, enhancing their fluidity, and consequently improving the oil extraction rate. However, higher temperatures are detrimental to the extraction of heat-sensitive compounds, and may activate some enzymes during the pretreatment process, leading to a decrease in oil quality. To date, most research on MAE has been conducted at the laboratory scale, and the cost-effectiveness of industrial-scale applications remains to be evaluated.

#### 2.1.6. Supercritical Carbon Dioxide Extraction (SFE-CO_2_)

SFE-CO_2_ involves contacting the oily raw material with supercritical carbon dioxide (SC-CO_2_), enabling the sequential extraction of components with different boiling points, polarities, and molecular weights. SC-CO_2_ is odorless, non-toxic, nonflammable, chemically inert, non-corrosive, and low-cost, which possesses the ability to dissolve triglycerides, fatty acids, and cholesterol. Notably, CO_2_ has a relatively low critical temperature and pressure (Tc = 31 °C and Pc = 7.38 MPa), allowing extraction to be conducted at moderate pressures ranging from 10 to 45 MPa and temperatures below 80 °C, making it suitable for extracting thermally unstable compounds [38,39]. Lin et al. utilized XSB as the raw material and carried out the extraction of XSBO by SFE-CO_2_. The optimal extraction conditions were determined as follows: pulverized particle size of XSB, 4 mesh (4.75 mm); extraction pressure, 25 MPa; extraction temperature, 45 °C; extraction time, 140 min. And a relatively high extraction rate could be achieved without the need to remove the shells [40]. Ma et al. optimized the SFE-CO_2_ process. The extraction temperature was set at 42 °C, the pressure was 28 MPa, and the extraction time was 192 min. Under these conditions, the extraction rate of XSBO was 81.22%, with a linoleic acid content of 42.30% and an oleic acid content of 33.76% [41]. Xie et al. developed a novel two-stage process for extracting XSB seed oil by utilizing SFE-CO_2_ followed by carbon dioxide-expanded ethanol (CXE). During the SFE-CO_2_ stage, the oil yield of XSB was 74.11%. Subsequently, through the extraction with CXE, an additional amount of oil accounting for 18.42% was obtained, resulting in a total oil yield of 92.53%. Furthermore, the lipid component analysis revealed that the content of nervonic acid in the oil obtained by CXE extraction was relatively high, reaching 4.12% [42]. Guo et al. compared the effects of various extraction methods, including pressing, AEM, and SFE-CO_2_ on the quality of XSBO. They found that the XSBO obtained by SFE-CO_2_ had a darker color and also had the highest acid value at 0.58 mg/g (calculated as KOH). This may be due to the fact that the process involved a relatively long period of high-pressure treatment, which led to an increase in free fatty acids [43]. Wang et al. compared the differences between SE, UAE, and SFE-CO_2_ in the extraction of XSBO. The results indicated that UAM achieved the highest extraction rate of 56.28%, but the XSBO extracted by SFE-CO_2_ had the widest variety of fatty acids, the highest content of linoleic acid, and the lowest content of erucic acid [44]. When extracting XSBO by supercritical extraction, the extraction parameters have a significant impact on the oil extraction rate. In terms of the oil extraction rate, the influence of the particle size of the material is complex. The extraction rate is relatively high when the particle size is around 20–30 mesh [38,39,40]. Increasing the extraction pressure within a certain range can increase the extraction rate, but when it exceeds a certain value, the effect is not good. The optimal pressure is approximately 25 MPa. As the extraction temperature rises, it promotes extraction, but at the same time, the change in the density of carbon dioxide has an opposite effect, and the optimal temperature range is 40–45 °C [42,43,44]. As the extraction time prolongs, the extraction rate first increases and then levels off. The optimal time is around 140–180 min. In terms of the composition of the oil, XSBO mainly contains various fatty acids such as linoleic acid and oleic acid, and the content of unsaturated fatty acids reaches 91.34%. Although the specific influence rules of each parameter on the fatty acid content are still unclear, supercritical extraction can effectively retain its unsaturated fatty acids and maintain its nutritional value.

SFE-CO_2_, as a green technology, has been widely applied in the extraction of functional edible oils due to its advantages such as non-toxicity, selective separation, and high efficiency. Compared with traditional techniques, SFE-CO_2_ yields a higher content of bioactive components and better physicochemical properties (color, aroma, acid value, and peroxide value). However, industrial production requires significant investment in equipment and operational costs. Currently, most applications are at the experimental or pilot scale.

#### 2.1.7. Other Extraction Methods

**Figure 2:**
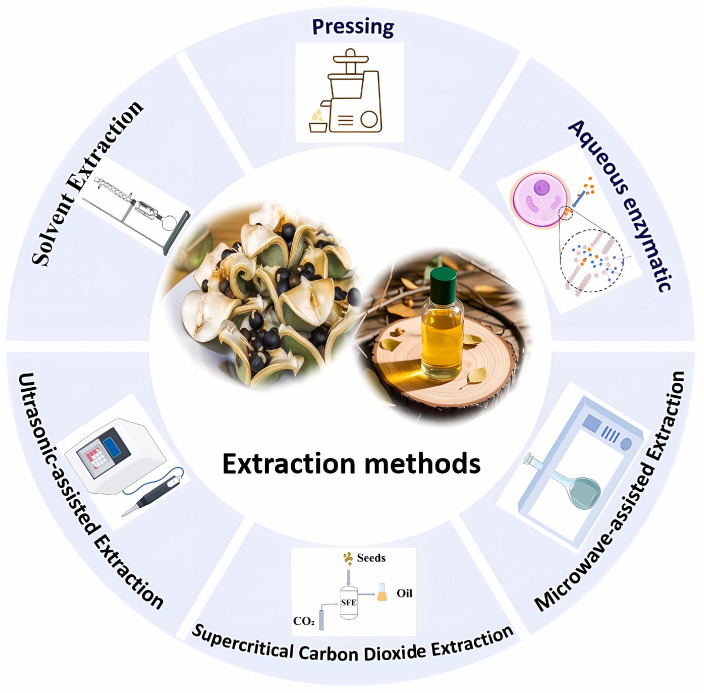
The extraction methods of XSBO.

Currently, the extraction of XSBO primarily relies on traditional methods (Figure 2). However, in recent years, some emerging extraction technologies have gradually been applied to the extraction of XSBO and its nutritional components. Examples include deep eutectic solvent extraction (DES), ionic liquid extraction (IL), and subcritical water extraction (SWE) [45,46,47]. These emerging methods not only improve the extraction yield and efficiency but also optimize the compositions of XSBO, thereby enhancing its functionality and application value. Nevertheless, research reports on these emerging extraction methods are relatively limited at present. Future studies could further explore the application of these technologies in the extraction of XSBO. By introducing more innovative extraction methods, it will be possible to gain a more comprehensive understanding of the characteristics of XSBO and further expand its potential applications.

### 2.2. Purification of Functional Components

XSBO is rich in various nutrients. However, there is currently a limited amount of research on the purification of functional components in XSBO. Here is a summary of the purification processes for nervonic acid (NA), linoleic acid (LA), and Phytosterols (PS) found in XSBO.

#### 2.2.1. Purification of NA

XSBO is rich in a unique fatty acid NA. Nervonic acid plays a crucial role in human physiological activities, particularly in the development and maintenance of the brain and nervous system, where it is indispensable. In many common vegetable oils, the content of NA is extremely low or even entirely absent. Relevant studies indicate that the content of NA in XSBO typically ranges from 1.5% to 3%. Jiang utilizes the HPLC method to create a fatty acid standard curve for quantitative analysis, and the content of NA in XSBO is measured as 2.08% [48]. Currently, the primary purification methods for NA in vegetable oils include molecular distillation [49], urea inclusion, solvent extraction [50], low-temperature crystallization, silver ion complexation [51], and chromatographic separation [52]. However, research on the purification of NA from XSBO is currently limited, with the main methods being molecular distillation, urea inclusion, and low-temperature crystallization.

Molecular distillation is a separation technique that operates under vacuum conditions. This method leverages the differences in the mean free path of molecular motion among various substances to achieve effective mixture separation. The mass of molecules is inversely proportional to their mean free path; lighter components volatilize first from the liquid and are enriched on the condenser tube, while heavier components cannot volatilize to the condenser tube and flow out along the wall, thus achieving separation of different substance components. The primary types of molecular distillation include falling-film, wiped-film, centrifugal, and improved-wiped-plate [53]. Chen et al. adopted a molecular distillation apparatus to refine the decolorized XSBO. With the content of NA in the heavy fraction as the main indicator, the impacts of the distillation temperature, feed rate, and wiper speed on the concentration of NA were explored. The results demonstrated that the content of NA in XSBO could reach 3.37% when the preheating temperature was 50 °C, the cooling water temperature was 10 °C, the pressure was 0.1 Pa, the distillation temperature was 225 °C, the feed rate was 900 g/h, and the rotational speed of the wiper was 350 r/min [54]. Gao et al. adopted the molecular distillation technique and set the process conditions as follows: the temperature was 130 °C, the pressure was 0.12 Pa, the rotational speed of the wiper was 270 r/min, and the feed temperature was 40 °C. The feed rates were set at 35 g/h, 30 g/h, and 25 g/h, respectively. The content of NA in XSBO was significantly increased from 3.1% to 30.4%. XSBO is not easily separated and extracted because of a relatively high boiling point. Ethyl esterification of XSBO can lower its boiling point, serving as an essential and critical step in the extraction of NA [55]. Zu et al. developed an efficient microwave-assisted transesterification (MAT) technique for preparing fatty acid methyl esters (FAMEs) from XSBO. Under the optimal MAT conditions of 500 W transesterification irradiation power, temperature 60 °C, 6 min, 1% wt catalyst, and a molar ratio of methanol/oil 6:1 (*v*/*v*), the maximum yield of FAMEs reached 96% [56]. Zhao et al. prepared fatty acid ethyl esters of XSBO by the transesterification method. By conducting a reflux reaction at a temperature range of 75–78 °C for 5 h, a ratio of ethanol to XSBO of 4:1, and a ratio of p-toluenesulfonic acid to XSBO of 1:10, XSBO fatty acid ethyl ester could be obtained. Subsequently, the prepared XSBO fatty acid ethyl ester was subjected to molecular distillation to enrich and obtain ethyl NA, with a content exceeding 50% [57].

The urea inclusion method is a technique that employs the low-temperature crystallization process of urea to incorporate straight-chain saturated fatty acids or monounsaturated fatty acids into its cage-like hexagonal crystals, and then form urea inclusion compounds that precipitate out, thereby facilitating the separation of fatty acids. Guo et al. utilized the urea inclusion method to enrich NA in XSBO. Under optimized conditions of a urea to fat ratio of 1:1, a material-to-liquid ratio of 1:10, a temperature of 10 °C, and a time of 8 h, the NA content in the enriched product reached 9.49%, which is approximately three times higher than before the inclusion, and the recovery rate of NA was 74.01% [58]. To improve the extraction efficiency and purity of NA, a strategy combining multiple purification technologies can be employed to further optimize the extraction process of NA. Gao et al. studied the extraction of NA from XSBO using the urea inclusion method, low-temperature crystallization method, and a combination of both methods. The results showed that the NA contents were 14.07%, 19.66%, and 40.17%, respectively. The combined treatment method increased the NA content by 12.62 times. Additionally, thermogravimetric analysis indicated that the material obtained through the combined treatment method exhibited stronger thermal stability [59]. Luo et al. used XSBO as the raw material and employed low-temperature crystallization, recrystallization, and the coupling method of recrystallization–column chromatography to purify NA from XSBO. The low-temperature crystallization process was optimized through single-factor and orthogonal experiments, and the optimal process obtained was as follows: the crystallization temperature was 5 °C, the time was 2 h, the solvent multiple was 1:9 (g/mL), and the ethanol concentration was 90%. Under these conditions, the purity of NA was 26.12%; after four recrystallizations, an NA product with a purity of 33.20% was obtained; by employing the coupling technique of recrystallization–column chromatography, an NA product with a purity as high as 85.76% was achieved [60].

In summary, the purification processes of NA mainly include low-temperature crystallization, urea inclusion, and molecular distillation. Among these, molecular distillation operates at a relatively low temperature, which effectively preserves the quality of the nutrients, but it requires multi-stage processing, and the operation process is complex. Low-temperature crystallization and urea inclusion are traditional extraction and separation processes that are simple to operate and relatively low in cost, but they have low efficiency and purity in extracting NA and may leave solvent residues. A strategy combining traditional processes with advanced technologies can be adopted to further improve the extraction rate and purity of NA.

#### 2.2.2. Purification of LA

XSBO is rich in unsaturated fatty acids, primarily oleic acid and LA [59]. LA is an essential fatty acid for the human body [61]. Studies indicate that LA can inhibit multiple types of chemically induced cancers, including gastric cancer, skin cancer, breast cancer, and colon cancer. Additionally, it may prevent arteriosclerosis, promote bone formation, and reduce cholesterol levels in both animals and humans [62]. Zhao used gas chromatography to analyze the LA in XSBO and found that the content of LA in the oil was 42.9% [52]. The main purification methods for LA include low-temperature crystallization, the urea inclusion method, lipase purification, high-speed counter-current chromatography separation, and molecular distillation. Xia et al. adopted the urea inclusion method to separate LA from XSBO. The optimal conditions were as follows: the ratio of urea to mixed fatty acids (MFA) was 1.5:1, the ratio of urea to ethanol was 6:1, the inclusion time was 18 h, and the inclusion temperature was 3 °C. The crude product obtained was detected by gas chromatography, and the mass fraction of LA was 92.17%, which was 53.42% higher than that of the original sample, and the yield of LA was 78.39% [63]. Zhao et al. employed the urea inclusion method to enrich LA in XSBO. Subsequently, the crude LA obtained was further separated and purified by high-speed counter-current chromatography (HSCCC). The results indicated that the combined use of the urea inclusion method and HSCCC for the separation and purification of LA was feasible, with the purity of LA reaching as high as 93.92% [64]. Yang et al. first enriched LA by the low-temperature crystallization method and then utilized the urea complexation method for further enrichment. Moreover, they optimized the optimal enrichment conditions by response surface methodology (the ratio of ethanol to free fatty acids was 3:1, with crystallization at −25 °C for 24.5 h, the ratio of urea to free fatty acid 1 was 2:1, the ratio of ethanol to urea was 6.6:1, and crystallization was carried out at −10 °C for 22.4 h), and the content of LA reached 97.10% [65].

#### 2.2.3. Purification of PS

PS are beneficial active compounds that exhibit multiple physiological functions, including immunomodulation, anti-inflammatory effects, antipyretic properties, antioxidant activity, and the ability to lower blood lipid levels. The common extraction methods for PS mainly include solvent crystallization, saponification, complexation, and column chromatography. XSBO contains a small amount of PS, approximately 1.09 g/kg [66]. Zhao et al. used the saponification method to extract PS from XSBO. The optimal technological conditions, when the total PS content in XSBO was 0.498%, were as follows: the solid-to-liquid ratio was 1:5 (g/mL), the saponification temperature was 72 °C, the saponification time was 2 h, and the amount of ethyl acetate was 200 mL; the crude PS were then purified by the solvent crystallization method, resulting in an increase in total sterol content from 14% to 55% [67].

Currently, there is limited research on the purification of functional components from XSBO. However, it can be observed that a single separation and purification method often fails to obtain high-purity nutrients due to its limitations (Table 1). To improve the extraction rate and purity of functional components in XSBO, the following measures can be taken: combining traditional technology and advanced approaches; processing samples through auxiliary technologies; and selecting green, non-toxic, and environmentally friendly solvents. The coupling of two or more methods can achieve complementary advantages and attain the desired separation effect.

## 3. Health Benefits of XSBO

### 3.1. Neuroprotective Effect

NA is a crucial component of nerve cells and brain tissue. It provides essential nutrition for nerve cells and effectively promotes the growth and repair of nerve fibers, playing a vital role in maintaining the health of the nervous system. However, the natural distribution of NA is relatively limited. Many common vegetable oils contain extremely low levels of NA, or none at all. Yang et al. conducted experiments that revealed the content of NA in XSBO to be 3.08%, while Agastache rugosa seed oil contained only 0.16%. And other common vegetable oils, such as peanut oil, soybean oil, rapeseed oil, corn oil, and olive oil, were found to contain no NA. The NA contained in XSBO has potential value for the prevention and treatment of neurodegenerative diseases such as demyelinating diseases (DD), Parkinson’s disease (PD), and Alzheimer’s disease (AD) [71]. Chen et al. investigated the effects on the fatty acids and blood lipids of cuprizone (CPZ)-induced demyelinating mice by ethyl NA from XSBO. The results indicated that the extract of ethyl NA from XSBO was capable of restoring and regulating the levels of NA in demyelinated mice, thereby serving as an exogenous supplement for NA in these animals. PD is a common neurodegenerative disease, mainly manifested as motor symptoms such as bradykinesia, rigidity, unstable gait, and tremor. At present, it is mainly treated with pharmacological therapy to relieve the symptoms, but there is no way to achieve a complete cure [72]. Hu et al. explored the improvement mechanism of NA extracted from XSBO on the antioxidant response and inflammatory response in PD mice. The results showed that the model and high-dose groups showed similar levels of apoptosis in behavior (*p* > 0.05). All three NA treatment groups showed decreases in IFs and increases in superoxide dismutase (SOD) and GSH-Px mRNA (*p* < 0.05). Therefore, NA can effectively alleviate the symptoms of PD [73]. Chen et al. investigated the key active components of XSBO for improving AD. The results indicated that NA could ameliorate the learning and memory dysfunction in rats with AD, reduce nerve cell damage, amyloid-β (Aβ) deposition, and tau protein phosphorylation in AD rats, enhance the antioxidant capacity of the rats and repair cholinergic deficits, and significantly decrease the levels of inflammatory factors [74]. XSBO has shown positive effects in improving neurodegenerative diseases such as DD, PD, and AD, and it holds the potential to become a new clinical treatment option. However, current research still has certain limitations. For example, there are differences between animal models and humans, and more research is needed to further validate its efficacy in the human body.

### 3.2. Antibacterial and Antioxidant Effects

XSBO is rich in various active components, including PS, flavonoids, and so on, which possess powerful antibacterial properties. These components can disrupt the bacterial cell membrane structure, thereby inhibiting the growth and reproduction of bacteria. In particular, PS, as an effective antibacterial agent, play a key role in the antibacterial effect of XSBO [75]. Some researchers have discovered that PS exhibit varying inhibitory effects on different bacterial strains. Liu et al. determined the size of the inhibition zones using the filter paper method and identified the minimum inhibitory concentration (MIC). They subsequently analyzed the antibacterial mechanisms of the PS extracted from XSB kernels by plotting growth curves, measuring the electrical conductivity, and assessing the leakage of intracellular contents. The results demonstrated that the PS in XSBO exhibited the strongest antibacterial effect against Staphylococcus aureus (with an MIC of 0.45 mg/mL), followed by Salmonella typhimurium (with an MIC of 0.50 mg/mL). The proposed antibacterial mechanism is that the PS from XSB kernels reduce the division rate of bacteria during the logarithmic growth phase, increase cell membrane permeability, and cause the leakage of intracellular electrolytes. This disruption affects bacterial growth and metabolism, ultimately leading to the death of bacterial cells [76].

XSBO contains various antioxidant active components, including PS, tocopherols, and saponins, which confer antioxidant properties to the oil. Zhang et al. evaluated the antioxidant activity of XSBO using free radical scavenging assays and β-carotene bleaching tests, yielding IC_50_ values of 0.151 g/mL and 0.195 g/mL, respectively. Furthermore, the results indicated that the antioxidant activity of XSBO demonstrated significant concentration dependence [77]. Some research has shown that the antioxidant activity of tocopherol in XSBO surpasses that of PS, but the antioxidant activity of ergosterol is stronger than that of tocopherol. The order of sterol free radical scavenging activities in XSBO, ranked from highest to lowest, is as follows: ergosterol, ergosta-7,22-dien-3-one, Δ5,24-stigmastadienol, stigmasterol-7,22-dien-3-ol, α-spinasterol, and Δ7-avenasterol [78]. Currently, there are relatively few reports on the antibacterial and antioxidant mechanisms of XSBO, and the research conducted thus far is only preliminary. Further in-depth studies are needed in the future to establish scientific evidence for developing it into medical and healthcare products.

### 3.3. Antitumor Effect

The various bioactive components in XSBO exhibit antitumor properties, including saponins, tocopherols, and PS [79]. They exert therapeutic efficacy against tumors through various mechanisms, such as inducing cell apoptosis, regulating the cell cycle, inhibiting the expression of oncogenes, or enhancing the expression of tumor suppressor genes. Yu et al. isolated and identified seven novel oleanane-type triterpenoid saponins, along with five known saponin compounds, from the oil residue of XSB seeds. Furthermore, they discovered that saponin compounds 7 and 9 demonstrated significant inhibitory effects on ten selected human cancer cell lines, which included HeLa (human cervical cancer cell line), BGC-823 (human gastric adenocarcinoma cell line), MCF-7 (human breast adenocarcinoma cell line), MDA-231 (human breast adenocarcinoma cell line), PC-3 (human prostate cancer cell line), HepG-2 (human liver cancer cell line), A549 (human lung adenocarcinoma cell line), A375-S2 (human melanoma cell line), Hep2 (human laryngeal cancer cell line), and HT1080 (human sarcoma cell line). Meanwhile, compounds 8, 10, and 11 exhibited moderate inhibitory effects on most of the cancer cell lines [80]. However, current research on the antitumor effects of XSB has primarily concentrated on its leaves and fruit husks, while studies focusing on the antitumor effects of XSBO remain relatively limited. Therefore, future research should intensify the investigation of the antitumor mechanisms of the bioactive substances in XSBO, providing a theoretical foundation for its clinical application.

### 3.4. Other Functional Activities

**Figure 3:**
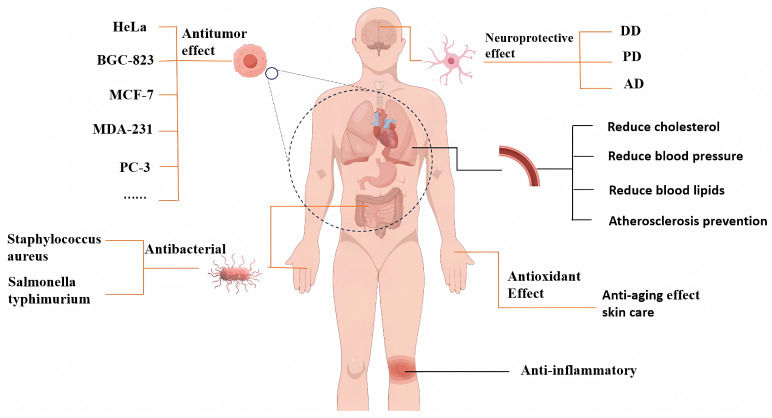
The health benefits of XSBO.

Apart from the functional activities mentioned above, XSBO also demonstrates additional beneficial properties. For instance, components such as unsaturated fatty acids, vitamin E, and PS in XSBO contribute to lowering cholesterol levels in the blood, protecting the cardiovascular system, and reducing the risk of cardiovascular diseases. Meanwhile, XSBO can improve gastrointestinal function, promote digestion and excretion, and help prevent constipation and intestinal diseases. Additionally, compounds like glutamic acid in XSBO have brain-enhancing properties and can improve memory. Furthermore, vitamins, polyphenols, and other trace elements present in XSBO exhibit antioxidant and anti-aging effects, providing cosmetic benefits such as skin care and hydration (Figure 3) [81].

## 4. Applications in Production and Life

XSBO has abundant nutritional components and multiple functionally active substances. It can be utilized in both the food and medicinal sectors, as well as in industrial production.

### 4.1. Food Field

With the improvement in living standards, people have increasingly become aware of the significant role that edible oils play in maintaining health. XSBO is transparent with a color ranging from light yellow to golden yellow, with a delightful taste as well as unique nutritional components and functional activities. It is recognized as a superior edible oil and is widely used in cooking, frying, baking, and other culinary applications. Although XSBO has a wide basis for consumption in folk practices, there is still very little research on its edible safety. Currently, there is significant public concern regarding the safety of edible oils. Fan et al. carried out acute toxicity tests, three genetic toxicity tests, and 30-day feeding experiments on XSBO. Neither poisoning symptoms nor deaths were observed among the mice throughout these experiments [82]. Lu et al. conducted gavage studies on pregnant SD rats with different doses of XSBO. It was found that XSBO showed no obvious maternal and embryonic toxicity or teratogenicity in SD rats [83]. The edible safety of XSBO has been preliminarily demonstrated, but long-term safety assessments of its consumption are still required along with further in-depth research to ensure reliability and safety. As an edible oil, XSBO undergoes a series of chemical and physical changes during the cooking process. Different cooking conditions have different impacts on the various active components of XSBO. The contents of NA, copper, zinc, manganese, and chromium in cold XSBO are all higher than those in hot XSBO, while the iron content, oxidation value, and saponification value are all lower than those in hot XSBO [84]. However, there is no significant difference in the fatty acid content between the two. Taking all these factors into consideration, XSBO can be consumed directly or the cooking conditions can be controlled within heating at 140 °C for no more than 5 min [85,86]. In addition, XSBO can also be used as an additive in various foods, such as flavored biscuits, candies, and beverages, providing nutritional and health benefits [87].

### 4.2. Medical Field

XSBO is widely recognized for its diverse pharmacological functions, including antioxidant, anti-inflammatory, antitumor, antibacterial, and immunomodulatory effects [6]. Notably, the fatty acid composition of XSBO is characterized by a high proportion of unsaturated fatty acids, primarily oleic acid and LA, with LA constituting approximately 40% of the total [88]. Oleic acid is known to promote vascular health and enhance metabolism, while LA is effective in reducing cholesterol levels, lowering blood pressure, and decreasing blood lipids, thereby helping to prevent atherosclerosis [89]. Consequently, XSBO is frequently utilized as a fundamental raw material in the production of medications such as “Linoleic Acid Dropping Pills”, “Yishouning”, “Linoleic Acid Microcapsules”, and other drugs. In particular, fatty acids also contain NA, a unique component not commonly found in other vegetable oils. It can reduce the damage to nerve cells in rats with AD and significantly reduce levels of inflammatory factors, which helps in improving AD symptoms and enhancing cognitive function in humans [74]. Lu et al. conducted a one-month gavage experiment on male mice using XSBO. The results demonstrated that the levels of 5-hydroxytryptamine (5-HT), acetylcholine (ACh), norepinephrine (NE), and dopamine (DA) in the homogenates of the hippocampus and cerebral cortex tissues of the mice in all groups were higher than those in the blank control group. Furthermore, with the increase in the concentration of XSBO, the contents of 5-HT, ACh, NE, and DA in the homogenates of the hippocampus and cerebral cortex tissues of the mice increased, while the activity of acetylcholinesterase (AChE) decreased. These results suggested that XSBO could effectively enhance the memory function of mice by improving the metabolism of monoamine and cholinergic neurotransmitters [90]. PS have a certain inhibitory effect on bacteria, such as Staphylococcus aureus, Escherichia coli, Salmonella, etc. [91]. Cao et al. analyzed the antibacterial characteristics of the PS crude extract against Escherichia coli, Bacillus subtilis, Mucor, and Penicillium. The results indicated that the PS in XSBO had a significant inhibitory effect on Escherichia coli and Bacillus subtilis, and the inhibitory effect on Escherichia coli was especially prominent [92]. XSBO has natural antibacterial properties and is a relatively good antibacterial agent. Therefore, it can be used as an antibacterial agent in combination with other drugs. Vitamin E has antioxidant and anti-inflammatory abilities and can delay aging and alleviate various inflammations. The content of vitamin E (with an average value of 757.86 mg/kg) in XSBO is much higher than that in peanut oil (413.5 mg/kg), rapeseed oil (595.9 mg/kg), sesame oil (320.0 mg/kg), and camellia seed oil (65.7 mg/kg) [79]. Therefore, XSBO is also used as a raw material to produce various drugs and health supplements for reducing inflammation. Other trace components in XSBO possess multiple active functions. For example, flavonoids exhibit anti-inflammatory and anti-rheumatic activities, and polyphenols possess antioxidant properties, capable of scavenging free radicals in the human body and improving skin condition, thereby achieving beauty and skin care effects. Furthermore, to improve the bioavailability of the active components in XSBO, it can be prepared into nano-emulsions, which enhance the stability and antioxidant properties of the oil. Qiao et al. conducted research on the preparation of XSBO nano-emulsions. The results indicated that the optimal process conditions were as follows: the whey protein concentration was 3.5%, the oil phase ratio was 9.8%, the shear speed was 15,000 (r/min), and the shear time was 3 min. Under these conditions, the prepared nano-emulsion exhibited an average particle size of 3 nm and possessed excellent stability and in vitro digestive activity [93]. However, there is limited research on delivery technologies for XSBO, which restricts its application in the medical field. XSBO holds broad application prospects in the medical field, but research on XSBO requires enhanced collaboration and communication with the medical community to facilitate its application and development in the medical field.

### 4.3. Industrial Field

In the field of bioenergy, with the continuous growth of global demand for clean energy and increasing concerns about the environmental issues associated with traditional fossil fuels, the biodiesel market has a promising future. Fu et al. used gas chromatography–mass spectrometry (GC-MS) to detect and analyze the content of unsaturated fatty acids in XSBO, which was as high as 94.36%, indicating that XSBO has the potential to be converted into high-quality biodiesel [94]. The biodiesel produced from it exhibits excellent combustion performance and low pollution emissions, aligning with the green transition needs of the transportation industry. Market demand in this sector is on an upward trend. The density of XSBO is 898.6 kg/m^3^ at a room temperature of 20 °C, while its kinematic viscosity is 7.91 mm^2^/s at 40 °C, its minimum freezing point is −4 °C, and its minimum pour point (with a blending ratio of B100) is −2 °C. Additionally, the cetane number of XSBO is 53, and its flash point is 121.2 °C, all of which meet the standards for biodiesel [95]. Therefore, XSBO serves as an excellent alternative to diesel. Li et al. took XSBO as the raw material and SZCe-HJ as the catalyst to optimize the preparation process of biodiesel by means of central composite design (CCD). The results showed that when the molar ratio of alcohol to oil was 8.21:1, the amount of catalyst was 2.66 wt.%, the reaction time was 8.65 h, and the reaction temperature was 56 °C, the yield of biodiesel reached its maximum value of 62.76% [96]. The production of biodiesel from XSBO has a relatively high yield, which can drive economic development and reduce the consumption of fossil fuels and pollution to the atmosphere. XSBO can also be used to make lubricating oil, but there are relatively few studies in the relevant fields. Hao et al. explored the research on the preparation of lubricating oil from XSBO. The results showed that when the epoxidation reaction temperature was 55 °C, the amount of concentrated sulfuric acid catalyst was 5% of the oil mass, and the reaction time was 8 h, the kinematic viscosity, density, and peroxide value of the prepared lubricating oil all reached their maximum values, which were 118.5 mm^2^/s, 0.9759 g/cm^3,^ and 9.8901 mmol/L, respectively [97]. In the chemical industry, XSBO can be used to synthesize products such as surfactants, coatings, and inks [15]. With the continuous increase in consumers’ preference for environmentally friendly and high-performance chemical products, chemical products made from XSBO, relying on their unique properties, are experiencing a gradual expansion in market demand.

However, there are numerous challenges in the process of large-scale commercialization. The cultivation scale of XSB determines the raw material supply base for its oil. On one hand, XSB exhibits strong environmental adaptability, capable of growing in arid and barren soils, theoretically possessing geographical advantages for expanding cultivation areas. However, its current cultivation range remains relatively limited, primarily constrained by insufficient scale in seedling breeding technology and uncertainties in growers’ expectations of economic returns. Breakthroughs in rapid propagation techniques for seedlings and improvements in standardized cultivation practices could potentially expand the cultivation scale rapidly, providing ample and stable raw material supply for industrial production. In terms of production costs, XSB has a long growth period from planting to fruiting, typically requiring 3 to 5 years to start bearing fruit and 8 to 10 years to reach full fruiting capacity [4,5,15,98]. During this period, significant human and material resources are required for management and protection, leading to high raw material costs. In the processing stage, the extraction and refining technologies for XSBO are complex, with substantial equipment investments, further driving up production costs. Environmental impact is also a factor that cannot be overlooked. Although the cultivation of XSBO contributes to ecological improvement, large-scale cultivation may have certain impacts on local biodiversity, necessitating assessment and regulation during development. Additionally, industries such as food and pharmaceuticals have extremely high requirements for the safety and quality stability of raw materials. Currently, the application standards for XSBO in these industries are not well established, and product quality varies significantly, posing a major obstacle to its widespread application. Only by comprehensively addressing these issues can the commercial feasibility of XSBO be clearly understood, and its large-scale commercialization in industrial fields be promoted.

## 5. Conclusions and Perspectives

XSBO is rich in nutrients and possesses multiple bioactive functions, showing great market development potential in the fields of food, medicine, health products, and industry. This review indicates that the extraction techniques for XSBO primarily include pressing, solvent extraction (SE), ultrasound-assisted extraction (UAE), aqueous enzymatic extraction (AEE), microwave-assisted extraction (MAE), and supercritical carbon dioxide extraction (SFE-CO_2_). However, the theoretical research on these extraction techniques is insufficiently in depth, resulting in relatively low oil yields and wasted resources. Currently, there are few studies focused on the purification of active substances such as LA, oleic acid, NA, vitamin E, and proteins in XSBO. XSBO has various bioactive functions, including neuroprotection, antibacterial, antioxidant, and antitumor effects. Nevertheless, the underlying mechanisms of these bioactivities have not been fully elucidated. Moreover, most of the research on its functional activities is based on in vitro experiments or animal simulation experiments. Although these experiments provide preliminary evidence, large-scale human clinical trials and research on relevant action mechanisms are lacking. In addition, due to its high content of unsaturated fatty acids, XSBO is prone to oxidation. However, there are few studies on the encapsulation technology of XSBO, which limits its application in food, medicines, and health products. Currently, the research on XSBO mainly focuses on the extraction technique. Further explorations are needed in the purification of functional components, the research on the action mechanisms of functional activities, encapsulation technology, and its application in medical and health products. Meanwhile, the research on the extraction and purification technology of XSBO still needs to be strengthened to improve its yield and quality and provide support for the large-scale production and applications of XSBO.

## Figures and Tables

**Table 1:** The oil yield and composition of different extraction methods.

Extraction Methods	Optimal Conditions	Extraction Yield and Main Compounds	References
Pressing	Pressure: 55 MPa ± 2 Kernel-to-shell ratio: 9:1 (g/g) Pressing time: 8 h	Oil yield: 40.44%	[17]
	Temperature: 20 °C Pressure: 55 MPa Pressing time: 6 h	Oil yield: 69.4% Linoleic acid: 42.93% Oleic acid: 29.06% Nervonic acid: 2.76%	[68]
	Pressure: 55 MPa-60 MPa Ratio of kernel to shell: 9:1 Pressing time: 8 h	Oil yield: 40.44%	[69]
Solvent extraction	Extracting agent: cyclohexane Liquid-to-solid ratio: 4:1 Extraction time: 3.0 h Extraction temperature: 70 °C	Oil yield: 30.02%	[20]
	Extracting agent: petroleum ether extraction temperature: 90 °C Liquid-to-solid ratio: 5:1 Extraction time: 10 h	Oil yield: 62.49%	[21]
Aqueous enzyme	Material-to-liquid ratio: 1:5 Enzymatic hydrolysis time: 5 h Enzymatic hydrolysis temperature: 50 °C Cellulase dosage: 1.5%.	Oil yield: 65.10%	[25]
	Temperature: 50 °C Neutral protease dosage: 3% Moisture content of the material: 12%	Oil yield: 74.82%	[26]
	Alcalase 2.4 L protease dosage: 0.02 mL/g Temperature: 55 °C Solid-to-liquid ratio: 1:6 Hydrolysis time: 4 h	Oil yield: 78.67%	[27]
	Ultrasound temperature: 50 °C Ultrasound time: 20 min Ultrasound power: 150 W Pectinase: cellulase ratio of 2:1 Enzyme addition amount: 1000 U/g	Oil yield: 71.5%	[28]
	Material-to-liquid ratio: 1:6 Temperature: 45 °C Alkaline protease (pH 7.0) dosage: 3.0% Cellulase (pH 4.5) dosage: 1.0% Reaction time: 8 h	Oil yield: 81.2%	[29]
Ultrasound-assisted extraction	Extraction solvent: petroleum ether Extraction temperature: 70 °C Extraction time: 34 min Liquid-to-material ratio: 7:1 (mL) Ultrasound power: 150 W	Single-pass oil yield: 58.92%	[32]
	Extraction solvent: petroleum ether Extraction temperature: 65 °C Extraction time: 30 min Liquid-to-material ratio: 9:1	Oil yield: 68.5%	[33]
	Ultrasound power: 150 W Extraction time: 120 min Cellulase addition: 0.05% NaCl addition: 0.10% Liquid-to-material ratio: 1:3.5 Enzymatic hydrolysis temperature: 30 °C	Oil yield: 89.85%	[34]
Microwave-assisted extraction	Extraction temperature: 75 °C Extraction time: 17 min Liquid-to-solid ratio: 20:1 Microwave power: 400 W	Oil yield: 58.85%	[38]
	Moisture content of raw material: 8.3% Kernel-to-shell ratio: 9:1 Microwave time: 5.4 min Microwave power: 960 W	Oil yield: 56.79%	[18]
	Microwave power: 300 W Liquid-to-material ratio: 4:1 NaCl concentration: 24 g/L Extraction time: 80 min	Oil yield: 56.79% Linoleic acid: 47.35% Oleic acid: 27.25%	[70]
Supercritical carbon dioxide extraction	Extraction temperature: 42 °C Pressure: 28 MPa Extraction time: 192 min	Oil yield: 81.22% Linoleic acid content: 42.30% Oleic acid content: 33.76%	[42]
	Two-stage process: combination of supercritical carbon dioxide (SC-CO_2_) and carbon dioxide-expanded ethanol (CXE)	Oil yield: 92.53% Nervonic acid content: 4.12%	[43]
	Extraction temperature: 45 °C Pressure: 30 MPaExtraction time: 150 min	Oil yield: 47.94% Linoleic acid content: 48.14% Oleic acid content: 28.76%	[44]
	CO_2_ cooling: −5 °C Supercritical CO_2_ flow rate: 18 L/h Extraction pressure: 28 MPa Extraction temperature: 40 °C Extraction time: 180 min	Oil yield: 78.6% Linoleic acid: 45.14% Oleic acid: 29.51% Nervonic acid: 2.75%	[29]

## Data Availability

No new data were created or analyzed in this study. Data sharing is not applicable to this article.
